# Influence of Preformed Antibodies in Liver Transplantation

**DOI:** 10.3390/jcm9030708

**Published:** 2020-03-05

**Authors:** Isabel Legaz, Francisco Boix, Manuela López, Rafael Alfaro, José A. Galián, Santiago Llorente, Jose A. Campillo, Carmen Botella, Pablo Ramírez, Francisco Sánchez-Bueno, José A. Pons, María R. Moya-Quiles, Alfredo Minguela, Manuel Muro

**Affiliations:** 1 Department of Legal and Forensic Medicine, Biomedical Research Institute (IMIB), Regional Campus of International Excellence “Campus Mare Nostrum”, Faculty of Medicine, University of Murcia, 30100 Murcia, Spain; isalegaz@um.es; 2 Immunology Service, Murcia Institute of Biosanitary Research (IMIB) and Biomedical Research Center in Liver and Digestive Diseases Network (CIBERehd), Virgen de la Arrixaca University Clinical Hospital (HCUVA), 30120 Murcia, Spain; franboix82@hotmail.com (F.B.); manolizar@hotmail.com (M.L.); raf.hellin@gmail.com (R.A.); diegoarmandogalian@hotmail.com (J.A.G.); sllorentev@telefonica.net (S.L.); josea.campillo@carm.es (J.A.C.); carmen.botellam@gmail.com (C.B.); rosa.moya2@carm.es (M.R.M.-Q.); alfredo.minguela@carm.es (A.M.); 3 Surgery Service, Murcia Institute of Biosanitary Research (IMIB) and Biomedical Research Center in Liver and Digestive Diseases Network (CIBERehd), Virgen de la Arrixaca University Clinical Hospital (HCUVA), 30120 Murcia, Spain; pablo.ramirez@carm.es (P.R.); sbuenof@um.es (F.S.-B.); 4 Digestive Medicine Service, Murcia Institute of Biosanitary Research (IMIB) and Biomedical Research Center in Liver and Digestive Diseases Network (CIBERehd), Virgen de la Arrixaca University Clinical Hospital (HCUVA), 30120 Murcia, Spain; jose.pons@um.es

**Keywords:** alcoholic cirrhosis, crossmatch, causes of death, donor-specific antibodies, liver rejection, forensic medicine, medico-legal autopsy, survival

## Abstract

The significance of human leukocyte antigen (HLA) matching and preformed donor-specific antibodies (DSAs) in liver transplantation remains unclear. The aim of this study was to analyze the presence of DSAs in a large cohort of 810 liver recipients undergoing liver transplant to determine the influence on acute (AR) or chronic liver rejection (CR), graft loss and allograft survival. DSAs were identified using complement dependent cytotoxicity crossmatch (CDC-CM) and multiplexed solid-phase-based flow cytometry assay (Luminex). CDC-CM showed that a 3.2% of liver transplants were positive (+CDC-CM) with an AR frequency of 19.2% which was not different from that observed in negative patients (−CDC-CM, 22.3%). Only two patients transplanted with +CDC-CM (7.6%) developed CR and suffered re-transplant. +CDC-CM patients showed a significantly lower survival rate compared to −CDC-CM patients (23.1% vs. 59.1%, *p* = 0.0003), developing allograft failure within the first three months (*p* < 0.00001). In conclusion, we have demonstrated a relationship between the presence of preformed DSAs and the low graft liver survival, indicating the important role and the potential interest of performing this analysis before liver transplantation. Our results could help to detect patients with an increased risk of graft loss, a better choice of liver receptors as well as the establishment of individualized immunosuppressive regimens.

## 1. Introduction

Acute humoral rejection in organ transplantation is generally a result of the presence, in the recipient, of preformed antibodies against donor´s human leukocyte antigens (HLA), referred to as donor-specific antibodies (DSAs) [1]. Preformed antibodies cause rejection by binding to class I HLA antigens expressed on the endothelium of vessels in the transplanted graft, resulting in the activation of the classic complement cascade, inducing thrombosis and infarction of the graft and as a result immediate extraction is necessary [2,3,4].

Complement dependent cytotoxicity crossmatch (CDC-CM) is a vital tool assessing the alloimmune response for a particular donor/recipient matching. A positive CM (+CDC-CM) against the donor in heart [5], lung [6] and pancreas [7] transplantation would usually mean that a particular matching should not proceed [8]. Also, the influence of DSAs has been observed in early and late graft loss [9].

In contrast to other types of organ transplantation, liver-transplant recipients used to be considered highly resistant to DSAs [5]. Besides, the association between progressive fibrosis and the presence of DSAs in paediatric and adult liver-transplant recipients [10,11], the impact of DSAs in short- and long-term liver transplant outcome remains poorly understood [12,13,14,15,16]. Nevertheless, increasing evidence suggests that DSA are associated with both acute and chronic liver allograft rejection [17,18,19]. For these reasons, it seems prudent to re-examine the impact of DSA on liver allograft structure and function, including short- and long-term liver transplant outcome. In this regard, recent studies have reported several genetic markers and soluble molecules as potential surrogate biomarkers for the outcome of liver transplantation [20,21,22,23] and thus, allosensitization could be equally important for an improvement in liver transplant outcome [24]. 

Therefore, the aim of this study was to analyze the presence of preformed DSAs antibodies, using CDC-CM and multiplexed solid-phase-based Luminex assay, in a large cohort of liver recipients undergoing liver transplant to determine the influence on acute or chronic liver rejection, graft loss and allograft survival.

## 2. Materials and Methods

### 2.1. Patient Enrollment and Data Acquisition

A total of886consecutivemedical records of liver transplant patients were recruited from1988 to 2014at the University Clinic Hospital Virgen de la Arrixaca (Spain) and analyzed retrospectively. Socio-demographic data (age, sex) andmain liver transplantation indications, post-transplant complications (AR and CR), immunological characteristics, causes of graft loss, as well as post-transplant graft survival at 5 years were studied. 

Deceased patients (*n* = 10), patients with graft loss (*n* = 7) in the first week after liver transplantation, patients in whom specific test could not be carried out (*n* = 59) (e.g., absence of serum sample from the recipient or/and sample for donor cell extraction) orAB0 incompatibility (*n* = 1) were excluded from this study. Finally, a cohort of 810 liver transplants was analyzed including donor DNA samples for HLA typing and a pre-transplant recipient serum sample for HLA antibody testing. All patients gave their informed consent for inclusion before they participated in the study. The study was conducted in accordance with the Declaration of Helsinki, and the protocol was approved in 1988 by the Ethics Committee of HUVA (PI19/01194).

### 2.2. Diagnostic Criteria of Liver Transplant Indications

Liver transplantation indications considered in this study were alcoholic cirrhosis with and without viral infection, viral cirrhosis by hepatitis C virus (HCV)and/or hepatitis B virus (HBV), hepatocellular carcinoma, fulminant hepatitis, autoimmune hepatitis and other less common end-stage liver diseases, as primary biliary cirrhosis, primary sclerosing cholangitis, cryptogenic cirrhosis, Wilson’s disease, amyloidosis and retransplant were also considered taking into account the respective clinical and pathological considerations. (Table 1). HCV and HBV pre-infection diagnosis was determined as described somewhere else [25].

### 2.3. Liver Function Status in Liver Transplantation Patients

Liver function status in liver transplantation patients was evaluated by the Child-Pugh score and Model for End-Stage Liver Disease (MELD) scoring system. All analytical values of patients on waiting lists for liver transplant were collected for these scoring systems. A biochemical test was conducted to determine the Child-Pugh scores and to classify patients from low to high severity of damage into 3 groups, A, B and C [26]. MELD score is generally used to measure the state of liver function in patients. MELD score was calculated accordingly using a mathematical formula composed of serum creatinine, total bilirubin, and international normalized ratio (INR) [27,28]. Therefore, patients were subsequently classified into 4 groups. High MELD values corresponded with more severe liver damage.

Cold ischemia time (CIT)is defined as the time in hours from donor liver cross-clamping to cold storage solution removal of the organ [29]. The degree of hepatic fibrosis in all patients included in this study was shown as F4 fibrosis (METAVIR score) at the time of inclusion in the liver transplant waiting list.

### 2.4. Immunosuppressive Treatment 

Triple immunosuppressive therapy (methylprednisolone, azathioprine and cyclosporine A (CsA)) was originally used. CsA was administered to achieve 200–350 ng/mL serum level until 1998, except in those cases with renal dysfunction or severe infection. Later, CsA was changed for tacrolimus as calcineuric immunosuppressant. The majority of transplants analyzed in our series used tacrolimus as a calcineuric immunosuppressant. The number of transplants where cyclosporine was administered was infamous compared to the rest. Thereafter, due to the increased risk of myelosuppression, azathioprine was substituted by mycophenolate mofetil (MMF). MMF was initiated at a dose of 1.5 g/day, starting within 12 h from liver transplant, and was adjusted according to leukocyte counts and gastrointestinal adverse effects. 

With respect to steroids, 1 g of methylprednisolone was given in the perioperative time, and was subsequently tapered off to 5 mg/day until the end of the first month. Steroids were generally withdrawn within the first year post-transplantation, as long as the liver function was stable. 

Tacrolimus-based protocol was also used (0.10–0.20 mg/kg/day). In the post-transplantation period, the dose was adjusted to maintain the target levels through blood levels. Antibody induction therapy was not routinely given in our patients.

All recipients with HBV were subjected to prophylaxis with hepatitis B immunoglobulin and a nucleoside analogue, such as lamivudine or entecavir. Recurrent HBV was serologically diagnosed by the detection of HBsAg antibodies and by the molecular detection of HBV DNA in the recipient sera.

### 2.5. Liver Rejection Diagnosis

The diagnosis of acute liver rejection(AR), confirmed by histological evaluation of graft biopsies was based on conventional clinical, biochemical and histological criteria. The severity of AR was graded according to the Banff classification [30,31]. In case of an AR episode, the rescue therapy was based on the administration of bolus of 500 mg of methylprednisolone and steroid-resistant cases with muromonab CD 3 (OKT3; Orthoclone; Ortho Pharmaceuticals, Raritan, NJ, USA). In this study, only liver recipients with AR developed within a 6-week period after transplant were included.CR diagnosis was based upon histological findings [32]. Patients with persistent liver rejection were treated with tacrolimus (FK506; 0.1 mg/kg per 24 h) or were re-transplanted. For patients who had undergone re-transplantation, only data regarding the first transplantation were used. For both types of liver rejection (AR and CR) a follow-up of 5 years was made after liver transplantation.

### 2.6. Determination of the Causes of Liver Was Graft Loss

The main cause of liver graft loss analyzed in all patients and collected from medical death certificates and/or medico-legal autopsy in those cases of sudden death to determine the cause and circumstances of death.

### 2.7. T-cell Complement-Dependent Cytotoxicity Crossmatch Technique (CDC-CM)

Recipient’s pre-transplant serum samples were drawn and utilized for systematic DSA screening by CDC-CM analysis. To this purpose, donor T lymphocytes were obtained from lymph nodes and enriched utilizing the nylon wool. Treatment of patient´s serum with dithiothreitol (DTT) was additionally performed [33,34]. In this study, the pre-transplant B cell CM was not considered.

### 2.8. DSAs Luminex Anti-HLA Antibody Screening

Pre-transplant serum was also retrospectively analysed for HLA antibody screening using multiplexed solid-phase-based microbeads array (Mix and Single Antigens Class I and II Kits, OneLambda, CA, USA), performed according to the manufacturers recommended procedure [33,34]. Luminex and CDC-CM results were compared. It ought to be notice, that the Luminex system may or may not recognize Immunoglobulin G (IgG) anti-HLA cytotoxic antibodies.

Serial dilutions were also performed in order to avoid an eventual prozone effect that could mask high-level antibodies. In this case, a pre-transplant serum was considered positive when its Panel Reactive Antibodies (PRA) value was higher than 0% (PRA ˃ 0%)and the mean fluorescence intensity (MFI) was higher than 1500 (MFI ≥ 1500).

The presence of DSA was determined by comparing particular HLA antibodies detected in the liver recipient serum with the liver donor HLA type. DSA to donor HLA-A, -B, -C, -DRB1, -DRB3, -DRB4, -DRB5 and -DQB1 mismatched antigens were analyzed. DSA were subsequently classified according to HLA antigen class as follows, class I only, class II only or both and stratified according to the cumulative normalised MFI of the first preformed DSA (negative, 1500–7000, 7001–10,000, ≥10,000).

### 2.9. Statistical Analysis

Demographic, clinical and immunological data were collected in a unified database (Microsoft Access 2.0; Microsoft Corporation, Seattle, WA, USA) and the analysis was performed using SPSS 22.0 (SPSS software Inc., Chicago, IL, USA). Quantitative variables were expressed as the mean ± SD and qualitative variables as a percentage. Pearson’s χ2 and two-tailed Fisher’s exact tests were used to compare categorized variables between different study groups and the two-sided.

Student’s *t*-test was applied to compare mean values. The primary study outcome was the 5-year graft survival in liver recipients with and without a positive CDC-CM. To this purpose, the correlation of CM data and the outcome of 810 liver transplants was assessed by the Kaplan-Meier method. Differences in graft survival at 3rd month and at 1st, 2nd, 3rd, 4th and 5th years post-transplantation were compered using the log-rank test. 

Furthermore, univariate and multivariable Cox proportional hazard models considering recipient and donor age, MELD score, Child-Pugh score, cold ischemia time, transplant indications, preformed DSA with MFI > 10.000 and previous liver transplantationwere also applied in order to confirm any potential positive risk factor associations with primary outcome. Results were expressed as the hazard ratio (HR) and 95% confidence interval (CI). *p* values < 0.05 were considered significant.

## 3. Results

### 3.1. Socio-Demographic and Clinical Characteristics

Socio-demographic and main clinical characteristics of total cohort (*n* = 810) of liver transplant are shown in Table 1. Overall, the mean age of total receptor was (56.1 ± 12.1; years ± SD) and in donor (52.0 ± 15.7; years ± SD). Statistically significant differences were found between both age groups (*p* < 0.0001). Men recipients were represented in 59% while women were represented in 40%. Out of 810 liver recipients, AR was reported in 27.4% patients, with a 78.8% of episodes occurring within the first month after transplantation. On the other hand, CR frequency was of reported in a 6.8% of cases (Table 1).

71.3% of patients had MELD values ranging from10 to 19, 15.5% had MELD values higher than 19 and 13.2% had MELD values lower than 9. On the other hand, most patients were classified as Child-Pugh B (53.1%) or Child-Pugh C (30.2%). The median of cold ischemia time (CIT) in our cohort was 7.6 h (5.0–8.5; min-max).

The main indication for liver transplantation was alcoholic cirrhosis (43%), viral cirrhosis (21.7%), and hepatocellular carcinoma (8.9%). The frequency of re-transplants in our cohort was 15.1% and the rest of liver indications are shown in Table 1.

### 3.2. Positive Anti-Donor Specific Antibodies (DSA) in the Main Indications for Liver Transplantation

From the 810 analyzed liver transplants with complete data for T-cell CM, only 26 recipients (3.2%) were transplanted with a positive donor-specific T-cell CM (+CDC-CM), whereas the rest were negative (96.8%). The main indication to liver transplant and its relationship with positive or negative T-cell CM (CDC+ or CDC-) was analyzed (Table 2). Interestingly, alcoholic cirrhosis’ patients were shown to be the higher percentage of CDC+ (42.3%), followed by viral cirrhosis (30.7%), re-transplants (15.4%) and fulminant hepatitis (11.5%). The rest of the indications for liver transplant were negative T-cell CDC-CM. Moreover, neither of the remaining social, demographic, or clinical data were significant amongst both study groups.

### 3.3. Pre-Transplant DSA and Their Influence in Acute or Chronic Rejection Development

As shown in Table 2, AR frequency was similar in +CDC-CM patients (*n* = 5, 19.2%) and negative CDC-CM patients (*n* = 174, 22.3%). There were no variations in the frequency of the AR episodes between both study groups (*p* = 1.000). The first AR episode developed within the first six weeks after liver transplant (mean, 14.8 days). Three alcoholic cirrhosis patients out of 26 with +CDC-CM developed AR episodes (11.5%). The remaining +CDC-CM patients with AR were viral cirrhosis (3.8%) and fulminant hepatitis (3.8%). No AR was observed in the rest of the indications for liver transplantation in +CDC-CM patients.

As shown in Table 2, however, the incidence of AR in these patients was not statistically different from that in patients with pre-transplant −CDC-CM (174 out of 784, 22.3%). Despite the lack of statistically significant differences, however, patients transplanted through +CDC-CM experienced fewer AR episodes than those showing a −CDC-CM (19.2% vs. 22.3%; *p* = 1.000). There was also no distinction between patients transplanted with positive or negative CDC-CM (data not shown) in the severity of the rejection episodes. Similarly, to what was seen with CDC, pre-transplant DSA results assessed by Luminex did also not show any relationship with AR (data not shown).

Likewise, the incidence of CR was also analyzed regarding T-cell CDC-CM results. Overall, CR incidence in our series was 7.2% and only two patients transplanted with +CDC-CM developed CR (3.5%). The only liver transplant indication with CR was AC without viral infection and viral cirrhosis. Pre-transplant DSA by Luminex did also not show any significant difference with CR (data not shown).

### 3.4. Causes of Graft Loss in Positive T Cell CDC Crossmatching

The causes of graft loss in patients with pre-transplant +CDC-CM were also examined (Table 3). As opposed to −CDC-CM patients, a statistically significant number of patients with +CDC-CM lost their grafts due to the development of sepsis (30.7% vs 8.8%; *p* = 0.0006). Other causes of allograft loss in +CDC-CM patients included multiorgan failure (19.2%); cerebral haemorrhage, cardiac complications, shock (7.7%), and renal failure (3.8%). On the other hand, the causes of allograft loss in −CDC-CM patients were sepsis (8.8%), multiorgan failure (7.0%) and cardiac complications (1.7%) amongst others (Table 3).

### 3.5. Post-Transplant Graft Survival in Liver Recipients with Positive and Negative T Cell CDC Crossmatching

**Figure 1:**
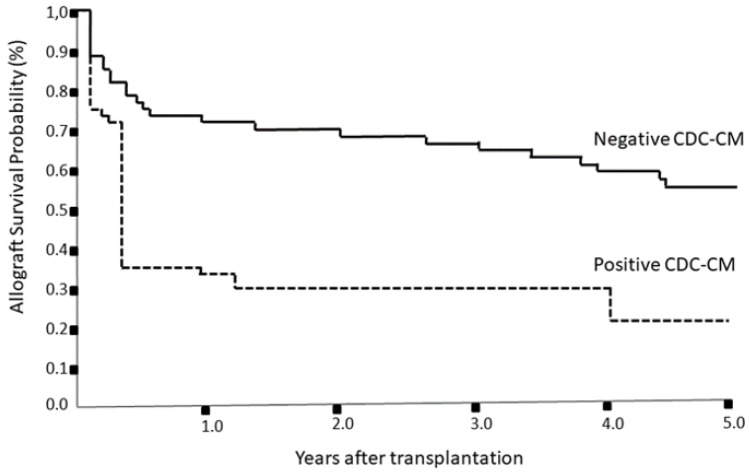
Kaplan–Meier allograft survival curves according to pre-transplant CDC-CM for liver transplant in the short and long term over 5 years. Continuous line represents negative CDC-CM group. Dashed line represents positive CDC-CM group. CDC-CM, complement dependent cytotoxicity crossmatching.

The relationship of CDC-CM data and the presence of DSA with the outcome of 810 liver transplants followed-up for at least 5 years was also analyzed. Interestingly, liver allograft survival was significantly lower in recipients with positive T-cell CDC-CM compared with those with negative CDC-CM across all the analysed post-transplant period of study time (Table 4, Figure 1).

Before the end of the first post-transplant year, most liver recipients with +CDC-CM suffered from allograft failure with an allograft loss rate of 69.2%. Because this highest incidence of graft failure shown within the first year post-transplantation, we further investigated the potential effect of the presence of preformed DSA as risk factor for graft loss during the first 12 months post-transplant. In logistic regression model, the presence of preformed DSA assessed by T-cell CDC-CM was shown to be the most significant risk factor to graft loss, whereby +CDC-CM study group showed the lowest graft survival rates in the 1st year post-transplantation (29.4% vs 72.3%, *p* < 0.0001).This loss took place mainly during the first 3 months after transplantation (34.6% vs 72.7%, *p* < 0.00001), confirming the main role that anti-HLA DSAs assessed by T-cell CDC-CM plays in the alloimmune response against the graft. The significance of the performance of this test prior to liver allograft transplantation as a predictive factor for the early graft tolerance rupture and organ failure. In consonance with results at 1st year, five-year allograft survival rates were seen to be significantly higher in the –CDC-CM study group as compared to the +CDC-CM group (59.1% vs 23.1%, *p* = 0.0003). On the other hand, no significant differences were found with regards graft survival between recipients without preformed DSA assessed by Luminex and those with maximum DSA MFI from 1.500–10.000 (Table S1). In addition, neither the class nor the strength of DSA reached statistical significance, except for an MFI >10.000 (*p* = 0.02).

In univariate analysis, no association was found between the main liver transplantation indications, donor age, recipient gender or immunosuppressive regimen (*p* > 0.05). Despite of this, all variables known to have clinical relevance were further included into the multivariate Cox analysis. Preformed DSA with a MFI > 10.000 (HR = 2.2; 95%CI = 1.7–5.3; *p* = 0.018) as well as any previous transplantation (HR = 1.02; 95%CI = 1.0–1.8; *p* = 0.043) were otherwise significantly associated with death within the first 12 months following transplantation (Table 5).

## 4. Discussion

In this retrospective study, we have analyzed, in a large cohort of liver recipients undergoing liver transplantation, whether the presence of preformed DSAs antibodies assessed by both CDC-CM and solid-phase based assay may be predictor factors to acute or chronic liver rejection, graft loss and allograft survival.

The liver exhibits intrinsic immune tolerogenic properties that contribute to acceptance when transplanted [35,36], and has been reported to be more resistant to the damage caused by HLA antibodies than other organs. The presence of preformed DSAs and its association with the development of graft rejection, graft loss, or even if different antibody concentrations exert different effects remains elusive [37]. 

There are fewer studies dissecting the relationship of CDC-CM and graft rejection or survival in liver transplant compare to other solid organs, despite the fact that their number is as of late expanding [38,39,40,41].

Our results show that the frequency of positive CM in our cohort (3.2%) was not significantly different to what had been reported in previous studies, where the ranges are between 3% and 11% [38,39,42,43,44,45], although some group has even reported until 28% [46]. 

With regards to preformed DSAs assessed by Luminex, 12% of our patients showed were seen positive, and this value is in concordance to other studies [47], although some authors has reported incidences up until 30% [46]. These discrepant results regarding the percentage of patients with +DSA assessed by Luminex might be explained because differences used in the MFI cut-off values (ranging 500 to 2000) amongst different Histocompatibility laboratories.

It is important to point out that the type and intensity of immunosuppression might have a detrimental effect in patient’s outcome due to DSA development. For instance, tacrolimus-free immunosuppression as well as MMF withdrawn protocols have been extensively reported as risk factors for de novo DSA development and acute rejection with no compromise in graft survival, respectively.

Our results show a strong association between the positivity for preformed lymphocytotoxic DSAs, detected by both CDC-CM technique and Luminex with a MFI level >10,000, with poor liver transplant allograft survival rates. In addition, from the first post-transplant year, a deleterious effect of positive CM on graft survival was observed. However, the acute and CR frequency was not different from that found in patients transplanted with a negative CDC-CM or pre-transplant DSA by Luminex.

With regards the assessment of pre-transplant cytotoxic antibodies assessed by CDC-CM, our results are similar to those reported by others showing that the presence of preformed DSA has no effect on the incidence of early AR in liver transplantation [38,43,44,48].

Other studies, on the other hand, have shown a significant impact of the pre-transplant +CDC-CM on the development of early graft rejection in liver transplant with organs from living or cadaveric donors [37,47,49,50,51,52]. 

Other reports of a higher number of AR episodes and a higher incidence of CR [53,54,55,56,57,58]. Therefore, this conflicting point remains unclear to date. 

Respect to CR, our results did not reveal an influence of the +CDC-CM, therefore our results are in accordance with others studies [38,43,47], however, other authors indicate otherwise higher incidence of CR in pre-sensitized HLA recipients [56,58]. However, the incidence of CR currently seen in liver transplant, appears to be declining gradually down to4% [57,59]. However, a previously published study reported the high prevalence of graft fibrosis and DSAs in late protocol biopsies [10]. 

Another important point is the type of IgG subclasses with regards antibody-mediated rejection (ABMR). IgG1 and IgG3 has been observed as the most representative DSA in patients with poor graft function, while patients with CR had a combination of subclasses of IgG, mainly immunoglobulin G2 and G4 (IgG2 and IgG4). By far, IgG1- and IgG3-associated ABMR have been linked with more acute phenotype, early presentation, rapidly graft dysfunction, positive C4d deposit, more responsive treatment and an early graft loss in in comparison with patients with DSAs of other IgG subclasses or without DSAs [14,60]. Perhaps the discrepancies found in the different studies might be due to the low number of patients studied.

On the other hand, the opinion about influence of liver allograft survival and the cross-matching are currently divided, since there was no difference in graft survival between transplants with and without CM in the earliest large series [61,62]. These observations were also confirmed by other studies [38,39,47]. However, our data showed that liver allograft survival in recipients with positive T-cell CDC-CM was significantly lower than recipients with negative CDC-CM, observing the same effect by other authors [19,43,56,63].

In our study, it should be noted that a large percentage of liver recipients with +CDC-CM had higher rates of early allograft failures mainly within the first 3 post-transplant months as well as at 1st year post-transplantation. In this sense, a previous study has shown that recipients with T-cell +CM-CM had significantly poorer outcomes than the -CM group [64]. Amongst these +CM recipients, 44% died and 85.2% revealed +C4d findings [64]. In other study, six out of the 10 patients with ductopenic rejection had circulating DSA and diffuse portal C4d deposit, three of whom developed unrelenting cholestasis, necessitating even specific antibody-depleting therapy to salvage the grafts [65].

Together with data from this study and other recent reports, it is suggested that the presence of preformed anti-HLA DSA antibodies in liver recipients may be associated with a higher degree of graft loss in adult recipients, where even some authors, like Groh et al., have strongly recommended CDC-CM testing to be always performed before liver re-transplantation [66].

This can easily be evaluated, as shown in the present work in a large series, with a pre-transplant CDC-CM and/or with pre-transplant DSA Luminex analysis and taking into account +DSA determination with high MFI (>10.000). The role of other possible additional tests (as i.e., C1q-DSA determination) should also be contemplated in further future studies we might performed, in similar manner to other studied organs [66,67,68]. 

Several authors have also shown that DSA (preformed or de novo) is an independent predictor of patient death in simultaneous liver-kidney transplantation [69]. Many reasons may explain the uncertain role of HLA antibodies in human liver transplantation as the central effect and problems like biliary sepsis, viral infection, systemic sepsis and recurrent disease, which are possibly a more important problem than rejection [5,70].

Besides, several immunological mechanisms have been proposed to explain the possible resistance of the liver to antibody-mediated immune injury, including the release of soluble HLA antigens and formation of immune complexes, the action of Kupffer cells against immune complexes, the double blood supply and single sinusoidal vasculature of the liver, and complement-mediated lysis deficiency [59]. On the other hand, other authors believe that this resistance could be due to the large size of the liver where antibodies spread in a large number of cells, thus increasing the encounter of two IgG molecules, which are necessary to fix the complement, less likely than in other organs like the kidney. They also suggest that due to liver regeneration capacity, antibody-induced damage decreases [5,12,71]. This effect of antibody resistance has also been observed in the post-transplant period [72].

Therefore, a clear picture for the particular role of the humoral alloresponse in liver transplantation is still absent. In this sense, very interesting reviews of the conflicting influence of HLA antibodies in liver transplant have recently been published by our group and others [73,74]. Other interesting point is the dilemma between preformed and/or *de novo* DSAs, in our present study, we have assessed the role of preformed DSA, but our next step will be to analyse the development of *de novo* DSA in post-transplant samples at different post-transplant times and their correlation with transplant outcome.

However, it is wise to recognize that performing CDC-CM might be highly time-consuming and so may hinder its prospective implementation in the Histocompatibility laboratory during liver transplantation on-call. In the same way, although a virtual cross-match could be beneficial, it would require donor HLA tissue typing delaying the transplant increasing otherwise the ischemia time. Our study is retrospective and seeks to analyze if its pre-transplant performance would have been useful. Obviously, these times can be shortened with greater rigor either at the extraction moment of pre-transplant samples to the Immunology laboratory, the use of HLA typing by real-time-PCR or the use of cross-match by flow cytometry. Likewise, shorter times might be achieved when conducting the CDC cross-match procedure. These points should be further clarified in the future.

Finally, our study has potential limitations. First, the low number of patients with +CM-CDC, but like other articles in the bibliography, this could make difficult the assumption of modify clinical protocols. Second, the impact that a retrospective study could have in the trustworthiness of the recorded clinical data. These two main points should be thoroughly address in a future prospective observational study.

## 5. Conclusions

In summary, our study found the presence of preformed cytotoxic anti-DSA antibodies as a predictive risk factor to lower liver allograft survival, indicating the important role of performing allogeneic CDC-CM prior liver allograft transplantation in order to predict early rupture of graft tolerance and organ failure. In addition, to our knowledge, our results add to the field of liver transplantation a potential immunological risk stratification protocol based on the level of MFI in retransplanted patients. In view of our results, the detection of antibodies before liver transplantation could help to detect patients with an increased risk of liver graft loss, a better assessment of liver receptors or the establishment of more appropriate and individualized immunosuppressive regimens.

## Figures and Tables

**Table 1:** Demographic data and main liver transplantation indications.

Total Number of Transplantations	810
Age recipient, (mean ± SD)	
Recipient	56.1 ± 12.1 ^a^
Donor	52.0 ± 15.7
Post-transplant liver rejection, *n* (%)	
Acute rejection (AR) ^b^	222 (27.4)
Chronic rejection (CR) ^c^	58 (7.2)
Recipient Gender, *n* (%)	
Male	478 (59%)
Female	332 (41%)
MELD score, *n* (%) *	
<9	107 (13.2)
10 to 19	578 (71.3)
20 to 29	111 (13.7)
30 to 39	14 (1.8)
Child-Pugh score, *n* (%) **	
A	135 (16.7)
B	430 (53.1)
C	245 (30.2)
CIT (h)	7.6 (5.0–8.5)
Transplantation indications, *n* (%)	
Alcoholic cirrhosis (AC)	351 (43%)
AC without viral infection	271 (33.4)
AC withviral infection ***	80 (9.9)
Viral cirrhosis ***	176 (21.7)
Retransplant	122 (15.1)
Hepatocellular carcinoma	72 (8.9)
Amyloidosis	25 (3.1)
Wilson’s disease	19 (2.3)
Fulminant hepatitis	16 (1.9)
Primary biliary cirrhosis	13 (1.6)
Cryptogenic Cirrhosis	9 (1.1)
Primary sclerosing cholangitis	4 (0.5)
Autoimmune hepatitis	3 (0.4)

N, number of individuals with a particular disease, AC, alcoholic cirrhosis; CIT, Cold ischemia time. h, hours, HBV, hepatitis B virus; HCV, hepatitis C virus., SD, standard deviation ^a^ Recipient and donor age was compared by the two-sided T-Student test, *p* < 0.0001.^b^ AR group and male or femalewere compared by the 2-sided Fisher’s exact test (*p* = 0.876; *p* = 0.763, respectively).^c^ CR group and male or female were compared by the 2-sided Fisher’s exact test (*p* = 0.351; *p* = 0.982, respectively).* Model for End-Stage Liver Disease (MELD) classification was considered taking into account bilirubin, international normalized ratio, and creatinine, where patients were classified into 4 groups based on theoretical mortality at 3 months [27]. ** Child-Pugh score based on 5 variables including serum levels of bilirubin and albumin, prothrombin time, ascites, and encephalopathy [26]. Based on the obtained values, patients were classified into low (Class A), intermediate (Class B), and high risk (Class C). Analytical values were obtained to get on the waiting list for liver transplantation. *** HBV or HCV infection. The mean values were analyzed (mean value ± SD) in all cases.

**Table 2:** Analysis of the main indications for liver transplantation and their relationship with T-cell Complement-dependent cytotoxicity crossmatch technique (CDC-CM) and graft rejection.

	+CDC CM Patients, 26 (3.2%)			-CDC CM Patients, 784 (96.8%)			
Main (LT) Indications, *n*	CDC+	Acute Rejection	Chronic Rejection	CDC-	Acute Rejection	Chronic Rejection	*p*
	*n* (%)	5 (19.2%) ^a^	2 (7.6%)	*n* (%)	174 (22.3%)	56 (7.14%)	
Alcoholic cirrhosis (AC), *n*	11 (42.3)	3 (11.5)	1 (3.5)	340 (33.2)	76 (22.4)	24 (7.0)	ns
AC without viral infection	5 (19.2)	1 (3.8)	1 (3.5)	266 (34.0)	59 (22,2)	19 (7.1)	ns
AC with HCV or HBV	6 (23.1)	2 (7.6)	0(0)	74 (9.43)	17 (23.0)	5 (6.7)	ns
Viral cirrhosis *	8 (30.7)	1 (3.8)	1(3.5)	168 (21.4)	38 (22.6)	12 (7.4)	ns
Retransplant	4 (15.4)	0 (0)	0(0)	118 (15.0)	25 (21,1)	9 (7.6)	ns
Hepatocellular carcinoma	-	-	-	72 (9.2)	18 (25.0)	6 (8.3)	ns
Amyloidosis	-	-	-	25 (3.2)	6 (24.0)	2 (8.0)	ns
Wilson’s disease	-	-	-	19 (2.4)	2 (10.5)	2 (10.5)	ns
Fulminant hepatitis	3 (11.5)	1 (3.8)	0 (0 )	13 (1.6)	3 (23.1)	1 (7.7)	ns
Primary biliary cirrhosis	-	-	-	13 (1.6)	4 (30.7)	0 (0)	ns
Cryptogenic Cirrhosis	-	-	-	9 (1.1)	1 (11.1)	0 (0)	ns
Primary sclerosing cholangitis	-	-	-	4 (0.5)	1 (25.0)	0 (0)	ns
Autoimmune hepatitis	-	-	-	3 (0.4 )	0 (0)	0 (0)	ns

* HBV or HCV infection. CDC, complement dependent cytotoxicity; HBV, hepatitis B virus; HCV, hepatitis C virus; LT, liver transplantation; AR, acute rejection; CR, chronic rejection; OR; Odd Ratio. Comparisons were made between + CDC-CM AR and –CDC-CM AR groups. ^a^ *p* = 1.000 OR = 0.835 (0.310–2.246); ns, no significant.

**Table 3:** Causes of graft loss in positive and negative T-cell CDC crossmatching.

	+CDC-CM Patients (N = 26)	−CDC-CM Patients (N = 784)		
Causes of Graft Loss	N = 20, *n* (%)	N = 160 *n* (%)	OR (95% CI)	*p* ^a^
Sepsis	8 (30.7)	69 (8.8)	6.908 (2.731–17.476)	**0.0006**
Multiorgan failure	5 (19.2)	55 (7.0)	-	ns
Cardiaccomplications	2 (7.7)	13 (1.7)	-	ns
Cerebral Haemorrhage	2 (7.7)	11 (1.4)	-	ns
Shock	2 (7.7)	9 (1.1)	-	ns
Renal failure	1 (3.8)	3 (0.4)	-	ns

N, total number of patients; *n*, number of patients with a particular cause of graft loss; CDC, complement dependent cytotoxicity; CI, confidence interval; OR, odds ratio. ^a^ Comparisons were made by the two-sided Fisher exact test. Significant *p* values are marked in bold. ns, not significant.

**Table 4:** Post-transplant liver graft survival frequencies in liver recipients with positive and negative T-cell CDC crossmatching.

				Allograft Survival, *n* (%)				
		Patients N = 810	3 months N = 579	1 year N = 575	2 years N = 530	3 years N = 491	4 years N = 486	5 years N = 469
**CDC-CM**	**−**	**784**	570 (72.7)	567 (72.3)	522 (66.6)	483 (61.7)	478 (61.0)	463 (59.1)
	**+**	**26**	9 (34.6)	8 (29.4)	8 (29.4)	8 (29.4)	8 (29.4)	6 (23.1)
**Log-rank (*p* values)**			**<0.00001**	**<0.0001**	**0.0004**	**0.002**	**0.003**	**0.0003**

N, total number of individuals of each group; *n*, number of surviving patients in each period of time. CDC-CM, complement dependent cytotoxicity crossmatching; (+), positive; (−), negative. Comparative table showing statistically significant differences between the liver graft survival and the positive or negative CDC crossmatch at different times. Significant *p* values are marked in bold.

**Table 5:** Values of multivariate Cox regression analysis respect to survival at first year.

Variable	HR	95% CI	*p* Value
Recipient age	0.7	0.9–1.1	0.058
Donor age	0.8	0.7–0.9	0.187
MELD score	1.2	1.0–1.3	0.052
Child-Pugh score	1.05	0.9–1.1	0.537
Cold ischemia time	1.04	1.0–1.2	0.520
Transplant indications	1.06	1.0–1.4	0.053
Preformed DSA with MFI > 10.000	2.2	1.7–5.3	**0.018**
Previous liver transplantation	1.02	1.0–1.8	**0.043**

HR, Hazard Ratio; MFI, Median Fluorescence Intensity; DSA, Donor-Specific Antibodies; CI, Confidence Interval. MELD, Model for End-Stage Liver Disease. Significant *p* values are marked in bold.

## Supplementary Materials

The following are available online at https://www.mdpi.com/2077-0383/9/3/708/s1, Table S1: Analysis of positive DSA antibodies by Luminex anti-HLA antibody screening showing different MFI levels.
