# Supplementary material for: Influence of Preformed Antibodies in Liver Transplantation
Source: J Clin Med. 2020 Mar 5;9(3):708. doi: 10.3390/jcm9030708 (PMC7141359; doi:10.3390/jcm9030708)
Supplement: Supplementary file 1 [file jcm-09-00708-s001.pdf]

Table S1. Analysis of positive DSA antibodies by Luminex anti-HLA antibody screening showing different MFI levels.

**DSA+, N=97 (12%), n (%)**	**MFI_{DSA}=1.500-7.000 n=44 (45.4%)**	**MFI_{DSA}=7001-10.000 n=27 (27.8%)**	**MFI_{DSA}>10.000 n=26 (26.8%)**
**HLA Class I**	15 (34.0)	11 (40.7)	10 (38.5)[a]
**HLA Class II**	11 (25.0)	4 (14.8)	5 (19.2)
**HLA Class I & II**	18 (41.0)	12 (44.5)	11 (42.3)

DSA, Donor-Specific Antibodies; MFI, Median Fluorescence Intensity.The value always corresponded to the first detected DSA.OR: odds ratio; CI: confidence interval. [a]Comparisons were made between HLA Class I with MFI= 1.500-7.000 and the same group with MFI>10.000. P = 0.045; OR = 0.323; 95% CI, 0.118-0.884).

Supplementary S1. Analysis of preformed DSAs Luminex anti-HLA antibody screening and their correlation with CDC-CM technique

Preformed DSAs assigned by Luminex technology were detected according to HLA type (HLA class I and/or class II) and cumulative MFI (Table S1). A total of 97 patients (12%) were positively detected by Luminex technology respect to total patients (n=810).

An increase statistically significant was observed in the detection of preformed positive DSAs HLA class I with MFI>10.000 (38.5%) respect to MFI = 1500-7.000 (34%; P = 0.045; OR = 0.323; 95% CI, 0.118-0.884). It should be noted that this is the only difference observed when comparing the rest of the groups analyzed.

It was analyzed if DSA previously detected with the CDC-CM technique, were correlated with data obtained by Luminex technology and MFI values, and its detrimental effect in patients undergoing liver transplantation.

The results were compared and it was observed thatseveral pre-transplant sera with -CDC-CM were DSA-positive when Luminex screening determination was applied (n=71; MFI=1500-10.000), but no notable influence was observed with liver transplant outcome. The concordance of results was observed only in with those cases with levels of MFI>10.000 in Luminex technology, this implies a concordance of 96% of the cases tested.

It should be noted that in the Luminex technology, only one recipient with positive anti-donor CDC-CM was negative, and this may be because this patient might have IgM antibodies (as DTT treatment was not performed in all patients during all years covering our retrospective study), or auto-antibodies, which are known to be able to result in a +CDC-CM, because this particular recipient is surviving without any problem at present.
